# Cultural Adaptations, Efficacy, and Acceptability of Psychological Interventions for Mental Health in Adults with Refugees and Asylum-Seeker Status: A Systematic Review

**DOI:** 10.1177/15248380241262262

**Published:** 2024-08-03

**Authors:** Lianne McDermott, Ikra Hameed, Alex Lau-Zhu

**Affiliations:** 1 University of Oxford, UK; 2 Oxford Health NHS Foundation Trust, UK; 3 Imperial College London, UK

**Keywords:** cultural adaptation, intergenerational trauma, mental health, psychological treatment, PTSD, ethnicity, refugees, asylum seeker

## Abstract

People with refugees and asylum seeker status (R/AS) have been forced to leave their home and resettle in new countries due to political unrest, conflict, and violence. This review aimed to describe the nature and extent of cultural adaptations to psychological interventions for adults with R/AS experiencing clinically significant psychological distress, and the acceptability and efficacy of these interventions. A search was conducted in October 2023 and February 2024 across five electronic databases: PsycINFO, Medline, Embase, PubMed, and Cochrane. Eligible studies were randomized controlled trials of psychological interventions conducted in any geographic context. Studies reporting on interventions with minimal adaptations only to facilitate treatment access, with no clear evidence for cultural adaptation, were excluded. Eighteen studies were identified, and cultural adaptations were described in line with the Ecological Validity Model. Studies investigating transdiagnostic interventions, cognitive behavioral therapy (CBT) interventions, and other psychotherapies were synthesized. Analysis and reporting of acceptability were limited across intervention groups, highlighting a need for more robust research in this area. CBT interventions and other psychological therapies were found to be most efficacious with moderate to large effects across validated psychological measures. Small to moderate effect sizes were observed across transdiagnostic interventions. The evidence quality was generally of some concerns. While the evidence requires further developments, the current review provides a timely synthesis of culturally adapted interventions for adults with R/AS to inform intervention development and clinical practice. Strengths, limitations, and recommendations for future research are discussed.

Emerging global crises have led to an increase in involuntary migrant movements, with the number of forcibly displaced persons exceeding 100 million in 2022 for the first time in history (UNCHR, 2022). Exposure to wars and armed conflicts (e.g., in Afghanistan, Ukraine, Sudan, Syria) forces people to leave their country of origin, and this situation is ever worsening. Specifically, the Israeli military operation in Gaza has led to the displacement of 2 million people as of March 2024 (House of Commons Library, 2024), and Russia’s military invasion of Ukraine has led to the displacement of at least 6.5 million people globally as of April 2024 (UNCHR, 2024). The 1951 Refugee Convention defined “refugees” as people unable to return due to fear of persecution for reasons of race, religion, nationality, or political, social status (McAdam, 2017). It is important to acknowledge that trajectories of people with R/AS are heterogenous. From a policy perspective, people with “refugee” status have been granted asylum in host countries, whereas “asylums seekers” are in the process of (or awaiting decision on) claiming asylum (Bradby et al., 2015). Post-migration stressors (Barbui et al., 2022), as well as dealing with the impact of traumatic events that initiated displacement, have been tied to high rates of post-traumatic stress disorder (PTSD), depression, anxiety, substance misuse, and sleep problems in adults with R/AS (Henkelmann et al., 2020; Patanè et al., 2022). Effective psychological interventions for this population are thus sorely required.

Developing psychological interventions in people with R/AS faces key conceptual and clinical challenges. Comorbidity rates across psychological disorders are elevated in R/AS populations (Belz et al., 2017; Im et al., 2022); thus, transdiagnostic approaches are particularly desirable. Expressions of psychological distress in R/AS populations are heterogenous, often characterized by locally salient somatic complaints and illness concepts (Morina et al., 2018; Rohlof et al., 2014). It has been argued that western diagnostic classifications may not best represent the mental-health symptom structures experienced across diverse refugee populations (Kronick et al., 2021). Cultural concepts of distress (CCD) appear to influence intervention effectiveness for people with R/AS (Heim & Kohrt, 2019). Psychological interventions for adults with R/AS are often developed in western contexts, raising questions about the cultural appropriateness of these for R/AS populations (Uphoff et al., 2020).

Systematic reviews and meta-analyses of randomized controlled trials (RCTs) have found cognitive behavioral therapy (CBT), eye movement desensitization and reprocessing (EMDR), and narrative exposure therapy (NET) to be overall effective for PTSD, depression, and anxiety in people with R/AS (Kip et al., 2020; Nosè et al., 2017; Thompson et al., 2018; Tribe et al., 2019; Turrini et al., 2019). However, findings are inconsistent; for example, NET was found effective for PTSD and/or depression in some studies (Kip et al., 2020; Nosè et al., 2017; Thompson et al., 2018; Tribe et al., 2019) but not others (Turrini et al., 2019). A better understanding of components or effective adaptations is needed.

## Culturally Adapted Interventions

Cultural adaptation refers to modification of evidence-based interventions to account for culture and context in a way that is consistent with an individual’s cultural patterns, meanings, and values (Bernal et al., 2009). Incorporating culturally relevant components has been found to enhance intervention efficacy for culturally diverse populations (Hall et al., 2016). There has been, however, a lack of consensus on how to classify adaptations. Modification to interventions could range from surface (e.g., language translation) to deep (e.g., shaping therapy components in line with individual’s cultural context) (Hwang, 2006).

Several adaptation frameworks have been developed (Day et al., 2023). The Ecological Validity Model (EVM) highlights eight components for cultural adaptation (Table 1; Bernal & Sáez-Santiago, 2006) and has been used to categorize adaptations in previous reviews (Chowdhary et al., 2014; Shehadeh et al., 2016; Wright et al., 2020). There is uncertainty about how the phenomenon of culture should be understood (Naem et al., 2019). On the one hand, culture refers to shared systems of understanding and engaging with the world, including political, economic, and other contexts that shape shared experience (Castellanos et al., 2020). However, it is important to extend beyond static manifestations of culture to encompass the spiritual, material, intellectual, and emotional features, encompassing art and literature, lifestyles, ways of living together, values, traditions, and beliefs (Kwiatkowska et al., 2002). Based on this, we argue that simply translating or adapting interventions to facilitate access—without additional considerations—would be considered a minimum standard delivery (as otherwise there would be no intervention at all).

**Table 1.** Framework for Cultural Adaptations (Bernal & Sáez-Santiago, 2006).

Component	Example Adaptation
Language	Intervention materials are made available in a client’s preferred language. Going beyond literal translation to incorporate colloquial expressions to replace technical terms, and use of cultural idioms of distress.
Persons	Cultural factors are considered in the therapist–patient relationship. Therapist and patient are acceptably matched, for example, emphasizing shared experiences and awareness of local customs. Demonstration of therapist cultural competence.
Metaphors	Use of culturally relevant materials. Use of stories and local examples with characters resembling the patient’s situation and background. Use of culturally relevant idioms and symbols.
Concepts	Constructs of theoretical model used in treatment are culturally relevant. Communication of the presenting problem and its constructs in a culturally appropriate. Addressing the somatic conceptualization of mental illness.
Goals	Development of personally and culturally relevant, client-derived therapy goals for example, family centered rather than individual goals.
Methods	Adapted procedures for achieving the treatment goals. Included simplifying the steps of treatment and reducing the focus on tasks requiring literacy such as reading and writing.
Context	Social, economic, and political context are considered and adaptations to increase access, for example, flexibility in scheduling sessions, inclusion of family members.
Content	Knowledge about values, customs, and traditions of a client’s cultural context. Content is adapted and integrated into all phases of a treatment process. Inclusion of local remedies and practices integrated into the treatment, for example, additional modules on spirituality.

## Aims of the Present Review and Comparison to Previous Reviews

There have been only two attempts to systematically review culturally adapted psychological interventions for adults with R/AS (Naseh et al., 2019; Wright et al., 2020). Despite the symptom heterogeneity and comorbidities in people with R/AS (Belz et al., 2017; Im et al., 2022; Morina et al., 2018), both reviews were limited to PTSD and/or depression outcomes. Neither study described theoretical rationale or empirical process for adaptation methods (Heim et al., 2021). Assessments of acceptability have also been neglected (Turrini et al., 2019). Critically, both reviews included studies with minimal adaptations to simply permit intervention access in refugee context (e.g., language, home delivery), without any additional evidence of adaptation to suit cultural patterns, meanings, and/or values.

Our overarching aim is to synthesize the literature on culturally adapted psychological therapies for adults with R/AS experiencing clinically significant psychological distress (beyond just PTSD/depression). Specific questions are:

1. What is the nature and extent of cultural adaptations to psychological interventions in this group?
2. How efficacious and acceptable are these adapted interventions?

## Method

This review was registered on PROSPERO (Reference: CRD42023385455) and adhered to the Preferred Reporting Items for Systemic Review and Meta-analyses (PRISMA) guidelines (Moher et al., 2015; Page et al., 2021).

### Search Strategy

A systematic review was conducted across five electronic databases: PsycINFO, Medline, Embase, PubMed, and Cochrane. A secondary search included reference lists of review studies and Google Scholar. The Population Interventions Comparison Outcomes Study Characteristics model (PICO) was used to frame search strategy (Richardson et al., 1995). Search strategy and inclusion/exclusion criteria are outlined in Table 2. A search (see Supplemental material Appendix A for search terms) was conducted in October 2023 and February 2024.

**Table 2.** PICO Search and Inclusion/Exclusion Criteria.

Component	Inclusion Criteria
Population	Study participants are above 18 years of age and with refugee or asylum-seeking status. Elevated psychological distress symptoms on at least one validated measure.
Interventions	Any psychological treatment with the aim of reducing symptoms of a psychological or somatic distress, as indicated by a validated measure. Active therapeutic modality (e.g., the intervention has clear theoretical underpinnings or an evidence base). Intervention in person, group, or individual, and can take place in any setting, by any trained provider (e.g., lay provider, psychologist).
Comparison	No intervention or control intervention (e.g., alternative intervention, treatment as usual, waitlist control)
Outcomes	Psychological and somatic symptoms as indicated by a validated measure.
Study	Randomized control trials conducted in any geographic context. No restrictions on publication date English language publications only A minimum of 12 participants in treatment arm to reduce risk of bias in results (Julious, 2005).
	Exclusion Criteria
	Intervention non-guided or digital Studies with adaptations to facilitate access only (e.g., translation) Studies that used a new intervention were not based on an existing intervention or psychological theory. Unpublished data, gray literature.

PICO = Population Interventions Comparison Outcomes.

### Selection Process

Studies were extracted into Endnote software for screening. Duplicates were removed. Titles and abstracts of remaining studies were screened by a first rater (LM), with 25% (*n* = 163) of these screened by a second rater (IH), with almost perfect agreement (κ = 0.93) (Cohens, 1960). The first rater screened for full texts, with 25% (*n* = 31) of these screened by the second rater, also with near perfect agreement (κ = 0.85).

### Data Extraction

Extracted data for each study included: author(s), publication year, sample size, population characteristics, intervention characteristics, and comparator type. For psychological distress outcomes, between group effect sizes with confidence intervals on validated outcome measures were obtained at baseline, post-treatment, and any follow-up data. In the absence of Cohen’s *d* effect sizes, means and standard deviations were obtained, and effect sizes were generated. Measures of acceptability included attrition and/or patient perspective via quantitative and qualitative analysis. Using the EVM (Bernal & Sáez-Santiago, 2006), cultural adaptations were described against eight dimensions (Table 1). Based on a recent framework for Reporting Cultural Adaptations in Psychological Therapies, information on the adaptation process (theoretical underpinning and formative research methods) was included (Heim et al., 2021).

### Quality Assessment

Quality assessment was performed using the Risk of Bias (ROB) tool from the Cochrane Handbook for Systematic Reviews of Interventions (Higgins et al., 2023). Each item was rated as low, high, or unclear bias across five domains: randomization, deviations from intended interventions, missing outcome data, measurement of the outcome, and selective reporting. IH cross-rated 20% of the included studies.

### Data Synthesis

Considerable heterogeneity across intervention theoretical models, setting, provider, and sample size meant that a meta-analysis was not indicated. Synthesis was conducted and reported following Cochranes guidelines (Cumpston et al., 2019) and Synthesis without Meta-Analysis guidelines (Campbell et al., 2020). Findings were grouped according to psychological intervention type, in line with a recent Cochrane review (Uphoff et al., 2020), interventions were grouped as “Transdiagnostic Interventions,” “CBT-based Interventions,” “Other Psychotherapies.” An overview of intervention grouping is shown in Supplemental material Appendix B.

To answer Question 1, cultural adaptations were synthesized descriptively using a table (Supplemental material Appendix E) and text summaries. To answer Question 2, a descriptive synthesis (Table 5) was utilized to illustrate acceptability and efficacy.

## Results

### Identification of Studies

**Figure 1.**
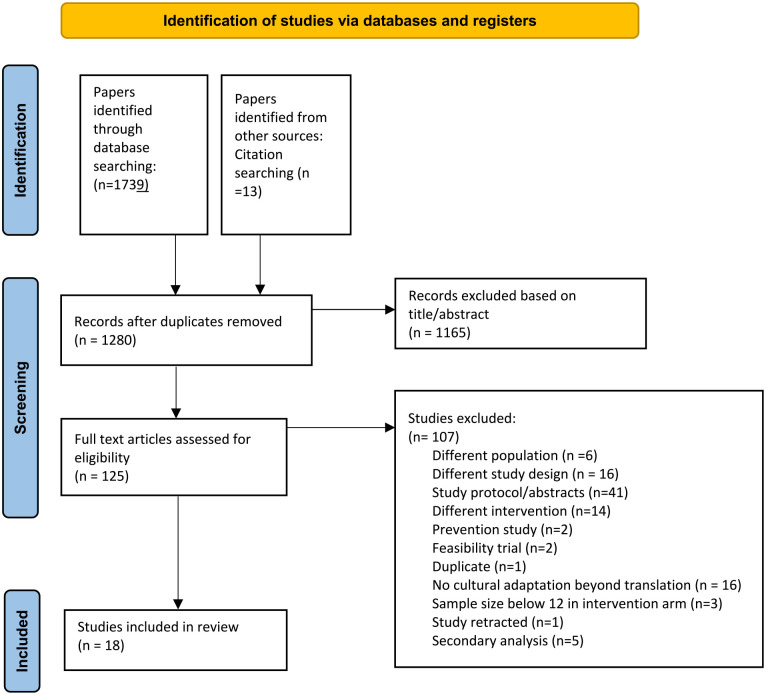
PRISMA of search strategy flowchart.

In total, 18 papers were identified for inclusion in the final review, comprising 2,269 adults with R/AS. An updated search yielded 85 studies, but none were appropriate for current review. A PRISMA flowchart is presented in Figure 1.

### Study Characteristics

Study characteristics and intervention type are summarized in Table 3. Eight studies were grouped as “transdiagnostic interventions,” six were “CBT interventions,” four were “other psychotherapies.” Studies included measures of PTSD (*n* = 17), depression (*n* = 15), anxiety (*n* = 12), somatic symptoms (*n* = 3), general symptoms of psychological distress (*n* = 6), adaptive stress (*n* = 1), and emotion regulation (*n* = 1). All studies described some demographics for adults (e.g., age and sex). Control groups included, waitlist (*n* = 8), treatment as usual (*n* = 6), delayed treatment (*n* = 2), or alternative active treatment (*n* = 2).

**Table 3.** Study Characteristics.

Intervention Group	Study	Country	Sample Size (IG, CG Number Recruited)	Population Age: M (SD), G: Female %, COO)	Intervention (D, MO, S, P)	EBT (Name of Intervention by the Authors)	Comparison Arm	Validated Measures
Group 1: Transdiagnostic Approaches	Acarturk et al (2022)	Turkey	*N* = 46 IG = 24, CG = 22	Age: 38.02 (10.88) G: 67.4% female COO: Syria	D: 5 × 60 m MO: Group S: Community P: Lay facilitators	PM+	TAU	PTSD: PCL-5, Depression and Anxiety: HSCL-25, Self-identified problems: PSYCHLOPS
	Bolton et al (2014)	Thailand	*N* = 347 IG = 182, CG = 165	Age: 34 (24–61 years) G: 63% Female COO: Burma	D: 10 × 60 m MO: Individual S: Homes, local ethnic clinics, community P: Lay facilitators	CETA	WLC	Depression: HSCL-25 PTSD: HTQ Anxiety: HSCL-25 anxiety subscale
	Bryant et al (2022)	Jordan	*N* = 410, IG = 204, CG = 206	Age: 40.03 (6.95) G: 70.2% female COO: Iraq	D: 5 × 2 hrs MO: Group S: refugee camp P: Lay facilitators	PM+	ETAU	Depression: HSCL-25 Anxiety: HSCL-25 PTSD: PCL-5 Self-identified problems: PSYCHLOPS
	de Graaff et al (2020)	Netherlands	*N* = 60 IG = 30, CG = 30	Age: 38.1 (12.2) G: 60% Female COO: Syria	D: 5 × 90 m MO: Group S: refugee camp P: Lay facilitators	SH+ (PM+ CAU)	TAU	Depression and Anxiety: HSCL-25 PTSD: PTSD symptoms check list Self-identified problems: PSYCHLOPS
	de Graaff et al (2023)	Netherlands	*N* = 206 IG = 103, CG = 103	Age: 26.5 (11.7) G: 38.3% Female COO: Syria	D: 5 × 90 m MO: Group S: refugee camp P: Lay facilitators	PM+	TAU	Depression and Anxiety: HSCL-25 PSTD: PCL-5 Self-identified problems: PSYCHLOPS
	Hasha et al (2022)	Norway	*N* = 76 IG = 38, CG = 38	Age: 33 (10.47) G: 36.8% Female COO: Syria	D: 6 × 150 m S: Community MO: Group P: Health professional and collaborating interpreters	TRT	AC: The Physiotherapy Activity and Awareness Intervention (PAAI)	PTSD: IES-R Psychological distress/Functioning: GHQ-12 Pain: BPI
	Greene et al* (2021)	Tanzania	*N* = 311 IG = 158, CG = 153	Age: 33.5 (SD = 9.0) G: 100% Female COO: Democratic Republic of Congo	D: 6 × NR (CPT), 2 × NR (Advocacy Counseling) MO: Group S: Refugee Camp P: Lay facilitators	CPT+ AC Nguvu	TAU	Depression & Anxiety: HSCL-25 PTSD: HTQ
	Tay et al (2020)	Malaysia	*N* = 331 IG: 170, CG: 161	Age: 30.8 (9.6) G: 28% Female COO: Chin, Rohingya, Kachin	D: 6 × 45 m weekly MO: Individual S: Community offices P: Non-specialist helpers trained for purpose of study	IAT	AC: CBT	Mental Health: RMHAP Adaptive Stress: ASI
Group 2: CBT	Eskici et al (2023)	Turkey	*N* = 23 IG = 12, CG = 11	Age: 35.1 (8.3) G: 100% Female COO: Syria	D: 7 × 120 m MO: Group S: community center P: Arabic speaking therapist	CBT CA-CBT	TAU	PTSD: HTQ Depression and Anxiety: HSCL-25
	Hijazi et al (2014)	USA	*N* = 63 IG = 41, CG = 22	Age: 48.2 (8.9) G: 55.6% Female COO: Iraq	D: 3 × 60–90 m MO: Individual S: Home, church, community center P: Therapist	NET	WLC	Depression: BDI-II, PHQ-15 PTSD: HTQ
	Hinton et al (2005)	USA	*N* = 40 IG = 20, CG = 20	Age: 51.8 (6.80) G: 60% Female COO: Cambodia	D: 12 × 60 m MO: Individual S: Community outpatient clinic P: Psychiatrist (author)	CBT CA-CBT	DT	PTSD: CAPS Anxiety: ASI Psychological Distress/Somatic complaints: SC90R
	Hinton et al. (2009)	USA	*N* = 24 IG = 12, CG = 12	Age: IG: 49.92 (9.23), CG: 49.08 (7.56) G: 60% Female COO: Cambodia	D: 12 × 60 m MO: individual S: Community outpatient clinic P: Psychiatrist (author)	CBT CA-CBT	DT	PTSD: CAPS Pain: O-PASS Flashback: O-FSS Cognitions: O-CCSS
	Kananian et al. (2020)	Germany	*N* = 24 IG = 12, CG = 12	Age: 22.1 (13.6) G: 0% Female COO: Afghanistan, Iran	D: 12 × 90 m (6 weeks) MO: Group S: University outpatient clinic P: Trained therapists	CBT CA-CBT+	WLC	Psychiatric: MINI Psychological distress/Functioning: GHQ-28 PTSD: PCL5 Depression: PHQ-9 Somatic: SSS-8 Emotion regulation: ERS.
	Shaw et al. (2019)	Malaysia	*N* = 29 IG = 20, CG = 9	Age: 33 (1.8) G: 100% Female COO: Afghanistan	D: 8 × NR MO: Group S: NR P: Lay facilitators, social worker	CBT CA-CBT	WLC	Depression: HSCL-25 PTSD: HTQ Anxiety: HSCL-25
Group 3: Other Psychotherapies	Acarturk et al. (2015)	Turkey	*N* = 29 IG = 15, CG = 14	Age: 35.27 (13.21) G: F = 75% female COO: Syria	D: 7 × 90 m MO: Individual S: refugee camp P: Psychologists	EMDR Standard EMDR Protocol	WLC	PTSD: IESR Depression: BDI:II
	Acarturk et al. (2016)	Turkey	*N* = 70 IG = 37, CG = 33	Age: 33.68 (10.56) G: 74% Female COO: Syria	D: 5 × NR (*M* = 4.3) MO: Individual S: refugee camp P: Psychologists	EMDR EMDR	WLC	PTSD: HTQ, IESR, Depression: BDI-II Anxiety: HSCL-25
	Aizik-Reebs et al. (2021)	Israel	*N* = 158 IG = 98, WLC = 60	Age: 31.8 (5.21) G: 46.2%, M = 43.8% COO: Eritrea	D: 9 × 2.5 hr MO: Group S: Community P: Mindfulness instructor + cultural mediator	Mindfulness based Intervention. MBTR-R	WLC	PTSD: HTQ Depression: PHQ-9 Anxiety: BAI
	Meffert et al. (2014)	Egypt	*N* = 22 IG = 13, CG = 9	Age: 31 (NR) G: 81% Female COO: Sudan	D: 6 × NR (2 × a week) MO: Individual S: Community offices P: Sudanese community trained for purpose of study	Interpersonal psychotherapy	WLC	PTSD: HTQ Depression: BDI-II

*Note.* AC = Active Control; ASI = Adaptive Stress Index; BDI-II = Beck Depression Inventory-II; BPI = Brief Pain Inventory; CA-CBT = Culturally adapted cognitive behavioral therapy; CAPS-5 = Clinician Administered PTSD Scale; CBT = Cognitive Behavioral therapy; CETA = Common Element Treatment Approach; CG = Control Group; CPT = Cognitive Processing Therapy; D = Duration (number of sessions × minutes); DT = Delayed Treatment; EMDR = Eye Movement Desensitization and Reprocessing; GAD-7 = Generalized Anxiety Disorder-7; HSCL-25 = Hopkins Symptom Checklist; HTQ = Harvard Trauma Questionnaire; IES-R = Impact of Events Scale-Revised; IG = Intervention Group; M.I.N.I. = MINI International Neuropsychiatric Interview; MBTR-R = Mindfulness based Trauma Recovery for Refugees; MO = Mode of delivery; NET = Narrative Exposure Therapy; NR = Not reported; P = Practitioner; PCL-5 = Post-traumatic Stress Disorder Checklist; PHQ-9 & 15 = Patient Health Questionnaire-9 & 15; PM+ = Problem Management plus; PSYCHLOPS = Psychological Outcome Profiles questionnaire; PTSD = Post-traumatic Stress Disorder; RMHAP = Refugee Mental Health Assessment Package; S = Intervention setting; SCL-90-R = Symptom Checklist-90-R; SH+ = Self Help plus; SSS-8 = Somatic Symptom Scale; TAU = Treatment As Usual; TRT = Teaching Recovery Techniques; WLC = Waitlist Control.

### Quality Assessment

ROB of all studies (Table 4) focuses on psychological distress outcomes. 17/18 (94.4%) of trials were classified as “some concerns” due to receiving at least one domain in this category. Strengths of the literature were: most studies (17/18; 94.4%) have “low risk” of randomization bias, deviation from interventions, and missing outcome data. Generally, a rating of “some concerns” across all trials was due to the use of self-report scales (e.g., rather than clinical interviews). Half of the studies (9/18; 50%) did not publish a protocol or intended analysis prior to trial, and as such classified as “some concerns.” The only Cluster-RCT (Green et al., 2021) was categorized as “high-risk” across recruitment domain due to recruitment process based on location (e.g., increasing risk of contamination within groups); “some concerns” across deviation from intervention domain, due to reported different intervention baseline conditions between groups; and “some concerns” for missing data due to baseline between-group differences that may have accounted for retention.

**Table 4.** Quality Assessment of Studies Using the Cochrane ROB 2.0 for RCTs and Cluster-RCTs.

Intervention Group	Study ID	Randomization	Identification of Participants (cRCTs)	Deviations From Interventions	Missing Outcome Data	Measurement of Outcome	Selection of Result	Overall
Group 1: Transdiagnostic interventions	Acarturk (2022)	Low		Low	Low	Some Concerns	Low	Some Concerns
	Bolton (2014)	Low		Low	Low	Some Concerns	Low	Some Concerns
	Bryant (2022)	Low		Low	Low	Some Concerns	Low	Some Concerns
	De Graff (2020)	Low		Low	Low	Some Concerns	Some concerns	Some Concerns
	De Graff (2023)	Low		Low	Low	Some Concerns	Low	Some Concerns
	Greene * (2021)	Low	High	Some Concerns	Some Concerns	Some Concerns	Some Concerns	High Risk
	Hasha (2022)	Low		Low	Low	Some Concerns	Low	Some Concerns
	Tay (2020)	Low		Low	Low	Some Concerns	Low	Some Concerns
Group 2: CBT-based interventions	Eskici (2023)	Low		Low	Low	Some Concerns	Some concerns	Some Concerns
	Hijazi (2014)	Low		Low	Low	Some Concerns	Some concerns	Some Concerns
	Hinton (2005)	Low		Low	Low	Some Concerns	Some concerns	Some Concerns
	Hinton (2009)	Low		Low	Low	Some Concerns	Some concerns	Some Concerns
	Kananian (2020)	Low		Low	Low	Some Concerns	Some concerns	Some Concerns
	Shaw (2019)	Some concerns		Low	Low	Some Concerns	Low	Some Concerns
Group 3: Other Psychotherapies	Acarturk (2016)	Low		Low	Low	Some Concerns	Low	Some Concerns
	Acarturk (2015)	Low		Low	Low	Some Concerns	Low	Some Concerns
	Aizik-Reebs (2021)	Low		Low	Low	Some Concerns	Some Concerns	Some Concerns
	Meffert (2014)	Low		Low	Low	Some Concerns	Some concerns	Some Concerns

*Note.* Study ID: First author and year of publication.

* Cluster RCT.

### Overview of Interventions

#### Transdiagnostic Interventions

*Transdiagnostic Interventions* (*n* = 8) included programs targeting common elements of multiple and co-occurring illnesses, which vary in terms of key mechanisms of actions and may combine several treatment approaches (Uphoff et al., 2020). Interventions include Problem Management Plus (PM+) (Acarturk et al., 2022; Bryant et al., 2022; de Graaff et al., 2020, 2023), combining psychoeducation, problem solving, and behavioral techniques; Teaching Recovery Techniques (TRT) (Hasha et al., 2022), including psychoeducation about intrusions, arousal and avoidance, and exposure; Common Elements Treatment Approach (CETA) (Bolton et al., 2014), combining psychoeducation, relaxation, behavioral activation, and exposure; Nguvu, based on Cognitive Processing Therapy (CPT) techniques (Greene et al., 2021), including advocacy support with psychoeducation, thought challenging, and relaxation; and Integrative Adaptive Therapy (IAT) (Tay et al., 2020), incorporating psychoeducation, problem-solving, exposure, emotion regulation, cognitive reappraisal, and meaning making. Of these, half took place in refugee camps (Bryant et al., 2022; de Graaff et al., 2020, 2023; Greene et al., 2021) or community settings (Acarturk et al., 2022; Bolton et al., 2014; Hasha et al., 2022; Tay et al., 2020). All *transdiagnostic interventions* were provided by lay facilitators, except the TRT intervention which was provided by trained professionals (Hasha et al., 2022). Lay facilitators included peer refugees and non-specialist charity workers. All studies reported facilitators were trained by certified trainers for 8 days, except in the CPT intervention (Greene et al., 2021) and CETA intervention where length of training was not stated (Bolton et al., 2014).

#### CBT Interventions

*CBT-based interventions* (*n* = 6) included studies testing interventions based on cognitive-behavioral theories of psychopathology (Eskici et al., 2023; Hinton et al., 2005, 2006; Kananian et al., 2020; Shaw et al., 2019), and one study testing NET, a type of CBT-based approach focused on embedding trauma exposure in an autobiographical context using a visual representation of a lifeline (Hijazi et al., 2014). Five studies took place in community settings (Eskici et al., 2023; Hijazi et al., 2014; Hinton et al., 2005; Hinton et al., 2009; Kananian et al., 2020); one did not record intervention setting (Shaw et al., 2019). All CBT-based interventions were provided by trained professionals.

#### Other Psychotherapies

*Other Psychotherapies* (*n* = 4) included one study utilizing a mindfulness-based approach or Mindfulness Based Trauma Recovery for Refugees (MBTR-R) (Aizik-Reebs et al., 2021), and one study utilizing Interpersonal Psychotherapy (IPT), a type of therapy developed to reduce impact of interpersonal difficulties (Meffert et al., 2014). Two studies utilizing EMDR (Acarturk et al., 2015, 2016), a trauma-focused treatment using bilateral simulations, theorized based on adaptive information processing (Shapiro & Forrest, 2001) and most recently on working memory theory (van den Hout & Engelhard, 2012). Two studies took place in refugee camps (Acarturk et al., 2015, 2016), while two took place in the community (Aizik-Reebs et al., 2021; Meffert et al., 2014). Two interventions were provided by trained professionals (Acarturk et al., 2015, 2016). One intervention was provided by both a trained professional and lay facilitator (Aizik-Reebs et al., 2021), and one study was provided by lay facilitators from the community who received 7 days of training (Meffert et al., 2014).

### Cultural Adaptations

Application of the EVM framework (Bernal & Sáez-Santiago, 2006) to selected studies was variable and shown in Supplemental material Appendix. Most domains were met by *transdiagnostic* and *CBT-based interventions*, with all *transdiagnostic interventions* adapting language, and most adapting across persons, metaphors, methods, goals, context, and content domains. All *CBT-based interventions* adapted across language and concept domains, with most *CBT-based interventions* adapting across metaphors, content, and context domains, half adapting across persons and concepts, and less than half adapting goals. *Other psychotherapies* were adapted to a lesser extent, with all studies adapting across language, person context and content domains, half of studies adapting metaphors, less than half of studies adapting methods, and no studies adapting goals and concepts.

#### Transdiagnostic Interventions

Levels of cultural adaptation were high across all *transdiagnostic interventions*, with all studies describing formative methods used to inform adaptations, including literature/desk reviews, focus groups, and workshops. Theoretical underpinnings for adaptations were not reported in majority studies (5/8; 62.5%). Of the studies that did, CDD provided theoretical rationale for adaptations (Bolton et al., 2014; Greene et al., 2021; Tay et al., 2020).

Half of the studies (4/8) described adaptation across all domains according to the EVM framework. Language adaptations were included in all studies, including adapting outcome measures to consider cultural idioms of distress. Therapist adaptations were found in majority studies (7/8; 94.4%) bar one (Hasha et al., 2022); for example, therapist-patient were matched by age, sex, and shared experiences through the use of lay facilitators.

Adaptation of metaphors was reported in most studies (7/8; 94.4%). All studies bar one (Greene et al., 2021), reported use of stories, images, and culturally salient examples to convey key principles. Cultural adaptation to therapy concepts was described in most studies (5/8; 62.5%). Examples included reconceptualizing adversity to include familial and social relationships (Acarturk et al., 2022; Bolton et al., 2014; Bryant et al., 2022; de Graaff et al., 2020, 2023), and prioritizing somatic symptoms and holistic support (Tay et al., 2020). Goals were adapted in all studies bar one (Greene et al., 2021), including changing the focus from individuals to others, for example, prioritizing family health over self, reducing impact of displacement on the collective, and building on existing strengths by emphasizing social/community support. Culturally adapted methods, aimed at maximizing acceptability, were described in majority studies (7/8; 94.4%) bar one (Bolton et al., 2014), reporting use of concrete strategies and reduced treatment elements.

Cultural context was considered in most studies (7/8; 94.4%) including adaptation to session timing and location (Bolton et al., 2014), condensed sessions over a shorter period (Greene et al., 2021), free childcare (Hasha et al., 2022), inclusion of family members (Bolton et al., 2014), culturally relevant case examples (Acarturk et al., 2022; de Graaff et al., 2020, 2023), and age or gender matched groups (Greene et al., 2021; Hasha et al., 2022). Adaptations to therapy content were included in most studies (6/8; 75%), including additional sessions to address group-specific concerns (Bolton et al., 2014; Greene et al., 2021); consideration of cultural beliefs and practices, for example, providing space for family engagement (Acarturk et al., 2022), or adaptation of relaxation components, for example, removing yoga in line with cultural preference (Acarturk et al., 2022; Bryant et al., 2022).

#### CBT-Based Interventions

Cultural adaptation was reasonably high across studies utilizing *CBT-based interventions*, with all studies reporting theoretical underpinnings and formative research used in adaptation process. Formative research included literature reviews, focus groups, adaptation workshops, and pilot studies. All studies described CCD as theoretical rationale for adaptations.

Studies described adaptation across three to seven domains. Language adaptations included adapting outcome measures to include cultural salient examples, as well as inclusion of cultural idioms of distress in all studies. For example, acknowledgment of cultural syndromes, for example, “Noba” indicating panic for Syrians (Eskici et al., 2023), and the Cambodian belief that “khyâl” overload occurs when blood rushes into the head (Hinton et al., 2009). Therapist adaptations were found in most studies (3/6; 50%). Studies matched therapist to patient by shared experiences, ethnicity (Shaw et al., 2019), and language (Hinton et al., 2005, 2009). Metaphors were adapted in all studies, including use of stories and proverbs to convey principles. Some studies adapted visualization exercises to include symbolic images, for example, lotus flower (Hinton et al., 2005, 2009), Persian garden (Kananian et al., 2020), or jasmine plant (Eskici et al., 2023).

Adaptation to concepts was described in all *CBT-based interventions*, including use of theoretical models to conceptualize cultural presentations of psychological distress. Goals were adapted in less than half the studies (2/6; 33.3%) (Hinton et al., 2005, 2009), and focused on reducing culturally specific symptoms, for example, “orthostatic panic attacks,” a type of panic attack generated by moving from sitting to standing, observed in Cambodian populations (Hinton et al., 2005, 2009). Methods were adapted in 3/6 (50%) studies, including simplifying treatment elements, and teaching cognitive flexibility through increased visualization in Cambodian populations (Hinton et al., 2005, 2009).

Some studies (3/5; 60%) reported adaptations to context (Eskici et al., 2023; Kananian et al., 2020; Shaw et al., 2019), including practical adaptations; for example, condensed sessions (Eskici et al., 2023; Kananian et al., 2020), or gender homogenous groups (Kananian et al., 2020). Adaptations to content were reported in all studies, and included focus on somatic symptoms, for example, using stretching to address mind-to-body connection (Eskici et al., 2023; Hinton et al., 2005, 2009; Kananian et al., 2020).

#### Other Psychotherapies

Cultural adaptation was reasonably high across *other psychotherapies*, with most studies (3/4; 75%) reporting formative process methods, including literature review, consultant groups/focus groups, adaptation workshops, and pilot studies (Acarturk et al., 2016; Aizik-Reebs et al., 2021; Meffert et al., 2014). Two studies (50%) reported stigma theory as rationale for adaptations made (Acarturk et al., 2015, 2016).

Studies described adaptation across four to five domains. Language adaptations were evident in all studies and included adapting outcome measures to include cultural idioms of distress. Therapist adaptations were found in all studies, for example, matching therapist to patient by sex, ethnicity, and/or shared experiences. One study included a cultural mediator alongside a trained professional (Aizik-Reebs et al., 2021). Metaphors were adapted in half of studies, including the use of religious imagery in a visualization exercise (Acarturk et al., 2015, 2016). Goals and concepts were not adapted. One study adapted methods, describing fewer elements, simplified language, and concrete strategies (Aizik-Reebs et al., 2021).

All studies reported adaptations to patient context, including session locations, condensed sessions, and free childcare. Finally, adaptations to content were described in all studies, including psychoeducation to address stigma (Acarturk et al., 2015, 2016), incorporation of beliefs and practices, for example, shared midsession meal (Aizik-Reebs et al., 2021), and culturally appropriate components, for example, role transitions (Meffert et al., 2014).

### Acceptability of Interventions

Table 5 shows key findings regarding attrition, retention, and acceptability. Overall reporting of acceptability was limited to retention and attrition rates across intervention groups. *CBT-based interventions* demonstrated the highest retention. Retention across *transdiagnostic interventions* and *other psychotherapies* varied, but no studies reported significant differences between intervention and control groups.

**Table 5.** Acceptability and Efficacy of Reviewed Studies.

Intervention Group	Study	Intervention	Acceptability	Efficacy	Effect Sizes
Group 1: Transdiagnostic Approaches	Acaturk (2022)	G-PM+	Retention in gPM+ was 75%. 25% (*n* = 7) dropped out of gPM+. Reason for drop out included lack of time, no approval from employer, responsibilities, and sickness. Semi-structured interviews with participants showed Syrian refugees had a positive view on the content, implementation, and format of gPM+.	A reduction in symptoms from post-assessment to 3-month follow up was observed, but there was no significant difference between GP+ and the CG for PTSD and self-identified problems. A small effect of GP+ on depression was observed. The authors note this study was not sufficiently powered to detect effect across all measures.	Depression and anxiety: HSCL-25: *d* = 0.23 PTSD: PCL-5: *d* = 0.01 Self-identified problems: PSYCHLOPS: *d* = 0.12
	Bolton (2014)	CETA	Retention in CETA intervention was 81.3%. 18.7% (*n* = 34) dropped out. Reasons for drop out: lack of time, a change in circumstances; one person died; and 15 could not be located. Drop out was greater within WLC (23.6%) compared to CETA group.	Compared to a WLC, CETA was effective in reducing symptoms of depression, PTSD, and anxiety in participants with R/AS. Effect sizes were large.	Anxiety: HSCL-25 (A): *d* = 0.79 Depression: HSCL-25 (D): *d* = 1.16 PTSD: HTQ: *d* = 1.19
	Bryant (2022)	PM+	Retention in PM+ was 82.4%. 14.7% dropped out prior to 6-week assessment, 17.6% dropped out prior to 3-month follow up. Reason for drop out: refusal, relocation, spouse disapproval, legal issues.	Measures taken at baseline, 6 weeks and 3 months of follow up indicated no significant difference across anxiety and PTSD measures between participants who received PM+ and participants in the TAU+. At 3 m follow-up, participants in PM+ showed greater reduction on depression scale, and self-identified problems than those receiving TAU+. Effect sizes were small.	Anxiety: HSCL-25 (A): *d* = 0.3 PTSD: PCL-5: *d* = 0.09 Depression: HSCL-25 (D): *d* = 0.4 Self-identified problems: PSYCHLOPS: *d* = 0.57
	De Graff (2020)	PM+	Retention in SH+ was 93.3%. Dropout rates were 3.16% in SH+ group. Reported reasons for non-attendance across assessments were “prefers to withdraw” (*n* = 3), “lack of time” (*n* = 3), “abroad/unavailable” (*n* = 1) and “no approval from spouse” (*n* = 1). No significant differences in rate of attrition between conditions Semi-structured interviews with PM+ participants and revealed positive perspectives on strategies, primarily due to positive therapeutic effect. A challenge to intervention adherence was “busy lives”	Participants were assessed at baseline; post-assessment and 3-month follow up. In the PM+ group, overall anxiety, and depression symptoms and PTSD symptoms decreased relative to participants who received TAU at 3-month follow up. Effect sizes were medium.	Depression and Anxiety: HSCL-25: *d* = 0.58 PTSD: PCL-5: *d* = 0.66 Self-identified problems: PSYCHLOPS: *d* = 0.81
	De Graff (2023)	PM+	Retention at 3-month follow-up was 85.4%, with data available for 84 participants (81.5%) in PM+. No significant differences in rate of attrition between conditions	Participants were assessed at baseline, post-assessment and 3-month follow up. At 3-month follow-up, PM+ had greater reductions on depression/anxiety relative to CAU. PM+ also showed greater reductions on PTSD symptoms. Effect sizes were small.	PTSD: PCL-5: *d* = 0.39 Depression and Anxiety: HSCL-25: *d* = 0.41
	Hasha (2022)	TRT	Retention of TRT was 68.42%. 31.6% dropped out. No significant differences in rate of attrition between conditions	There was no main effect of TRT intervention and the control group on measures of PTSD (IESR) and pain symptoms (BPI) were not significant. There was a medium effect on general mental health (GHQ-12) which decreased significantly only in the TRT intervention group during the 6-week period. Cohens *d* calculated from means and standard deviations provided by authors.	PTSD: IES-R: *d* = −0.16 Pain: *d* = 0 General Mental Health: GHQ-12: *d* = −0.84
	Greene (2021)	Nguvu	Retention of Nguvu group was 58.2%. Attendance was lower in women experiencing high IPV and more frequent sexual IPV. 11.6% of participants were lost to follow up. No significant differences in rate of attrition between conditions. Interviews with Nguvu participants found format and content of the Nguvu intervention was generally acceptable. The environment was described as collaborative and supportive (Greene et al., 2022).	This study found lower symptoms of depression, anxiety, post-traumatic stress in the Nguvu condition relative to the TAU at endline. Between-group differences revealed non-significant and very small effect sizes. Authors note this study was not sufficiently powered due to challenges with recruitment.	PTSD: HTQ: *d* = −0.12 Anxiety: HSCL-A: *d* = −0.09 Depression: HSCL-D: *d* = −0.12
	Tay (2020)	IAT	Retention of the IAT group was 97.6%. 2.4% (*n* = 4) dropped out. Dropout rate explained by relocation or resettlement in a third country.	Both IAT and CBT participants reported significantly lower scores on all primary mental health outcomes at 6 weeks, and at 12-month follow-up, compared to baseline, but treatment arms did not differ significantly from each other. Effect sizes were very small.	PTSD: RHMAP: *d* = 0.08 CPTSD: RHMAP: *d* = −0.07 MDD: RHMAP: *d* = −0.07 CDRS: RHMAP: *d* = −0.16
Group 2: CBT-based Interventions	Eskici (2023)	CA-CBT	Retention of the CA-CBT intervention was 90.7%. There were only two dropouts (8.3%) from CBT condition and one from control condition.	CBT had a large effect on PTSD and small effect for anxiety and depressive symptoms.	PTSD: HTQ: **d** = 1.17 Anxiety and Depression: HSCL-25: *d* = .40
	Hijazi (2014)	NET	Retention of the NET intervention was 95.1%. 4.9% (*n* = 2) dropped out. Post-study questionnaire: The mean reported satisfaction with brief NET was very high, and significantly higher than the reported benefit of treatment.	Compared to WLC, brief NET reduced post-traumatic stress and depression symptoms after 2 months, with medium-sized effects. Symptoms continued to decrease to the 4-month assessment; the WLC symptoms improved at 4 months, eliminating the difference between conditions. No significant between groups differences were observed at 4m follow up.	2-month follow-up: PTSD: HTQ: *d* = −0.48 Depression: BDI: *d* = 0.46
	Hinton (2005)	CA-CBT	100% retention and completion.	Compared to WLC, patients improved on all measures. Between-group comparisons indicated benefit over waitlist control on measures of PTSD, anxiety, and somatic symptoms, with very large effect sizes.	Anxiety: ASI: *d* = 3.78 PTSD: CAPS: *d* = 2.17 Somatic Symptoms: SC90-R: *d* = 2.77
	Hinton (2009)	CA-CBT	100% retention and completion.	The patients randomized to CBT had much greater improvement than patients in the WLC condition on measures of PTSD. After receiving CBT, the CG improved on this measure. Tailored measures to capture culturally specific symptoms indicated significant improvement for CBT group compared to CG. Effect sizes were very large.	Emotion Regulation: ERS: *d* = 2.53 PTSD: CAPS: *d* = 1.98
	Kananian (2020)	CA-CBT	Retention of the CBT group was 91.7%. 1 dropped out due to time constraints.	The CA-CBT+ intervention produced improvement in general psychopathological distress, symptoms of depression, somatic complaints, quality of life, and emotion regulation, and moderate effect sizes regarding PTSD symptoms, compared to the CG.	General Health: GHQ-28: *d* = 3.0. PTSD: PCL-5: *d* = 0.7 Depression: PHQ-9: *d* = 1.5. Somatic Symptoms: SSS-8: *d* = 1.8 Emotion Regulation: ERS: *d* = 2.2
	Shaw (2019)	CA-CBT	Retention of the CBT group was 80%. Attrition not explicitly reported.	From baseline to post-treatment assessments, initial intervention participants experienced significant declines in emotional distress, anxiety, depression, and post-traumatic stress. Comparing the treatment groups to the WLC revealed large effect sizes.	Anxiety: HSCL-25 (A): *d* = 2.31 Depression: HSCL-25 (D): *d* = 2.42 PTSD: HTQ: *d* = 2.07 Mental Health: RHS-15: *d* = 2.14
Group 3: Other Psychotherapies	Acarturk (2015)	EMDR Protocol	100% retention and completion for EMDR sessions.	EMDR group had significantly lower trauma scores at post-treatment as compared with the wait-list group. The EMDR group also had a lower depression score after treatment as compared with the wait-list group. These findings were maintained at 5-week follow up. However, the authors did carry out follow up assessment with the CG.	PTSD: IES-R: *d* = *1.78* *Depression*: *BDI-II: d* = *0.51*
	Acarturk (2016)	EMDR R-TEP	Retention of EMDR group was 75%. 25% (*n* = 12) dropped out. Reason for drop out: refusal of entering treatment (*n* = 7), moving location out of camp (*n* = 5). No significant differences in rate of attrition between conditions	EMDR group had significantly reduced PTSD and depression scores at post-treatment, and at 5-week follow-up compared to the control group. Results supported the efficacy of EMDR in reducing symptoms of PTSD and symptoms of depression. Cohens *d* calculated from means and standard deviation reported by authors. Effect sizes were moderate to large.	5-week follow up PTSD IES-R: *d* = 1.11 HTQ: *d* = −0.96 *Depression* BDI: *d* = −0.68 Anxiety and Depression HSCL-25: *d* = −0.71
	Aizik-Reebs (2021)	MBTR-R	Retention of MBTR-R group was 66.6%. 32.7% (*n* = 15) dropped out of trial. No significant differences in rate of attrition between conditions.	Participants assigned to MBTR-R demonstrated significantly lower levels of PTSD, depression symptoms, and anxiety symptoms relative to the waitlist control group. This was evident at post-intervention and at 5-week follow-up. Effect sizes were moderate to large in magnitude.	PTSD: HTQ: *d* = 0.91 Depression: PHQ: *d* = 0.81 Anxiety: BAI: *d* = 1.00 5-week follow-up PTSD: HTQ: *d* = 0.87 Depression: PHQ: *d* = 0.70 Anxiety: BAI: *d* = 0.67
	Meffert (2014)	IPT	Retention of IPT group was 90.9%. 9.1% dropped out of group. One participant withdrew because her husband forbode her to continue. One dropped out due to time constraints. No significant differences in rate of attrition between conditions.	IPT was successful in decreasing symptoms of PTSD and depression compared to WLC, with large effect sizes. For the IPT group, mean PTSD symptoms on the HTQ decreased by approximately 40%, compared with a decrease of approximately 9% in the CG. Mean depression symptoms on the BDI–II decreased by 63% in the IG, compared with a decrease of 16%, in the CG. Cohens *d* calculated from change scores and standard deviations reported by authors.	PTSD HTQ: *d* = 2.155 Depression BDI-II: *d* = 1.568

*Note.* AC = Active Control; ASI = Adaptive Stress Index; BDI-II = Beck Depression Inventory-II; BPI = Brief Pain Inventory; CA-CBT = Culturally adapted cognitive behavioral therapy; CAPS-5 = Clinician Administered PTSD Scale; CBT = Cognitive Behavioral therapy; CETA = Common Element Treatment Approach; CG = Control Group; CPT = Cognitive Processing Therapy; DT = Delayed Treatment; EMDR = Eye Movement Desensitization and Reprocessing; GAD-7 = Generalized Anxiety Disorder-7; HSCL-25 = Hopkins Symptom Checklist; HTQ = Harvard Trauma Questionnaire; IES-R = Impact of Events Scale-Revised; IG = Intervention Group; IPT = Interpersonal Psychotherapy; MBTR-R = Mindfulness based Trauma Recovery for Refugees; NET = Narrative Exposure Therapy; PCL-5 = Post-traumatic Stress Disorder Checklist; PHQ-9 & 15 = Patient Health Questionnaire-9 & 15; PM+ = Problem Management plus; PSYCHLOPS = Psychological Outcome Profiles questionnaire; PTSD = Post-traumatic Stress Disorder; RMHAP = Refugee Mental Health Assessment Package; SCL-90-R = Symptom Checklist-90-R; SH+ = Self Help plus; SSS-8 = Somatic Symptom Scale; TAU = Treatment As Usual; WLC = Waitlist Control.

### Transdiagnostic Interventions

All studies testing *transdiagnostic interventions* reported on retention and attrition, with some studies (3/8; 37.5%) reporting on acceptance via qualitative analyses (Acarturk et al., 2022; de Graaff et al., 2020; Greene et al., 2021). Acceptability across interventions according to attrition and retention rates varied from lower retention (Greene et al., 2021; Hasha et al., 2022), to high (de Graaff et al., 2020; Tay et al., 2020). No studies reported significant differences for attrition between intervention and control groups. There was limited reporting of qualitative and quantitative analysis of acceptability, although some studies (3/8; 37.5%) found treatment format and content were generally acceptable (Acarturk et al., 2022; de Graaff et al., 2020; Greene et al., 2021).

#### CBT-Based Interventions

All studies testing *CBT-based interventions* reported on attrition and retention, with one study reporting on patient acceptability via quantitative analysis (Hijazi et al., 2014). Rates of retention were relatively high across all *CBT-based interventions*, ranging from 80% (Shaw et al., 2019) to 100% retention (Hinton et al., 2005, 2009). For the one study reporting quantitative measure of acceptability, satisfaction was high (Hijazi et al., 2014). Therefore, *CBT-based interventions* were found to be acceptable for people with R/AS, but further analysis is required.

#### Other Psychotherapies

Both studies testing *other psychotherapies* reported on attrition and retention, with no studies reporting on patient acceptability via qualitative or quantitative analysis. Rates of retention ranged from 66% (Aizik-Reebs et al., 2021) to 75%–100% (Acarturk et al., 2015, 2016; Meffert et al., 2014). For the studies with higher attrition, control group attrition rates were comparable. Therefore, *other psychotherapies* were acceptable for people with R/AS according to attrition rates, but more data are required.

### Efficacy of Interventions

Table 5 shows this review’s findings regarding efficacy. Cohen’s *d* effect size was used to indicate intervention benefits. All *CBT-based interventions* and *other psychotherapies* demonstrated moderate to large effects on at least one validated measure of psychological distress. Most *transdiagnostic intervention* studies found small effects on at least one measure; however, findings across transdiagnostic approaches were mixed.

#### Transdiagnostic Interventions

A majority (5/8; 62.5%) of studies found effects for *transdiagnostic interventions* on at least one validated measure of psychological distress. Two studies found anxiety, depression, and PTSD symptoms decreased in Syrians who received PM+, with small to moderate effect sizes (de Graaff et al., 2020, 2023). A large effect on depression, anxiety, and PTSD was observed for participants who received a course of CETA (Bolton et al., 2014). Another study found no significant effect of PM+ on PTSD and anxiety, but a small to moderate effect on measures of depression and self-identified problems (Bryant et al, 2022). One study assessing TRT with people from Syria and found no significant effect on PTSD and pain symptoms, but a large reduction in general mental health symptoms (Hasha et al., 2022). Two studies were not sufficiently powered to test effects due to under recruitment (Greene et al., 2021) and pilot design (Acarturk et al., 2022). A small effect of IAT was observed in refugees from Chin, Rohingya, and Kachin in Malaysia, but treatment arms did not significantly differ from each other (Tay et al., 2020). Taken together, effects of *transdiagnostic interventions* were mixed, potentially influenced by heterogenous settings and methodological limitations.

#### CBT-Based Interventions

Regarding efficacy of *CBT-based interventions* all studies found a meaningful effect of CBT on psychological measures. Two studies found CBT to be effective reducing symptoms of PTSD, anxiety, and somatic symptoms (Hinton et al., 2005), and symptoms of emotion dysregulation and PTSD with large effect sizes (Hinton et al., 2009). Another study found CBT had a large effect on PTSD and moderate effect on depressive symptoms (Eskici et al., 2023). Moderate effect sizes for PTSD, and large effect sizes for symptoms of psychological distress, depression, somatic complaints, and emotion regulation were observed (Kananian et al., 2020). CBT was found to significantly reduce emotional distress, anxiety, depression, and post-traumatic stress with large effect sizes (Shaw et al., 2019). A study investigating NET found reduced PTSD and depression symptoms after 2 months, with small to moderate-sized effects, but no significant differences at 4-month follow up due to improvements in control group (Hijazi et al., 2014). Taken together, *CBT-based interventions* were effective for reducing symptoms for people with R/AS, but longer-term research is required.

#### Other Psychotherapies

Regarding efficacy of *other psychotherapies*, all studies found intervention effects on at least one validated measure of PTSD, depression, or anxiety. One study investigating the effect of MBTR-R found significant reductions with moderate to large effect sizes across depression, PTSD, and anxiety measures (Aizik-Reebs et al., 2021). Another study found IPT significantly decreased symptoms of PTSD and depression, with large effect sizes (Meffert et al., 2014). Two interventions found EMDR had a moderate to large effect on reducing PTSD and depression symptoms (Acarturk et al., 2015, 2016). Thus, *other psychotherapies* were effective in reducing symptoms of psychological distress across a range of measures for people with R/AS.

## Discussion

There is an urgent need for effective and appealing mental health interventions for people with R/AS. Thus, we sought to describe the nature and extent of cultural adaptations to psychological interventions offered to adults with R/AS, and the acceptability and efficacy of interventions. Regarding cultural adaptation, *transdiagnostic approaches* and *CBT-based interventions* were most adapted, with all transdiagnostic and majority CBT approaches describing adaptations across most domains of the EVM framework (Bernal & Sáez-Santiago, 2006). *Other psychotherapies* were adapted to a lesser extent, describing adaptations across approximately half of domains.

Most studies had a bias associated with subjective self-report measures and single blinding, which is difficult to avoid in low-resource contexts where extensive diagnostic interviews with trained raters are often unfeasible. With this limitation in mind, there was evidence of moderate to large effect sizes for psychological distress according to validated measures for *CBT-based interventions* and *other psychotherapies*. There was weaker evidence for *transdiagnostic approaches*, although most of these studies reporting modest effects on at least one validated measure of psychological distress, which may provide evidence for the truly transdiagnostic nature of interventions (Schäfer et al., 2022). Very few reported on acceptance beyond attrition rates, hindering our analysis of acceptability. Nevertheless, no studies reported significant differences for attrition rates between intervention and control groups across interventions. Critical findings of this review are summarized in Table 6.

**Table 6.** Critical Findings.

Area	Finding
Study heterogeneity	There was considerable heterogeneity across studies regarding intervention setting, provider, and control type, thus limiting validity of conclusions regarding intervention effects.
Cultural Adaptation: Process, Nature, and Extent	Most studies included in this review utilized formative research methods to inform adaptations. Theoretical underpinning of adaptations was reported in majority of studies. Cultural adaptations included “language,” use of cultural idioms of distress; “contextual,” modified timing and settings; “content,” incorporation of beliefs, practices, and rituals; “persons,” matched therapist to patient by sex, experience, or age; “metaphor,” use of culturally salient stories/images; and “concept,” redressing conceptualization of adversity to include familial/social relationships. “Goals,” that is, changing solution from individual to others, and “methods,” that is, use of concrete strategies and reduction of treatment elements, were adapted to a lesser extent across studies.
Acceptability	Very few studies have reported on acceptance beyond attrition, hindering analysis of acceptability across and within intervention groups.
Efficacy	CBT interventions and other psychotherapies were found to be efficacious with moderate to large effects across measures of psychological distress. Mixed evidence was found to support transdiagnostic interventions for people with R/AS, with most studies reporting modest effects on at least one measure.

We went beyond describing adaptations, as in past reviews (Naseh et al., 2019; Wright et al., 2020), and assessed for evidence of best practice in cultural adaptation, namely considering theoretical underpinnings and formative research methods (Heim et al., 2021). Theoretically driven approaches appropriately consider the psychological literature when understanding what to adapt and why, while formative research facilitates identification and evaluation of local and cultural need (Heim & Kohrt, 2019). We found theoretical underpinning reported in just over half the studies with most describing ethno-psychological theories such as CCD and few describing stigma theory as a basis for adaptations. Formative research methods, however, were described in most studies. Reporting of theory and formative research allows for transparency and replicability and can inform studies testing mechanisms of change in R/AS populations.

### Methodological and Clinical Implications

To facilitate comparability, we grouped studies according to intervention type based on a Cochrane umbrella review of psychological interventions for people with R/AS (Uphoff et al., 2020). Within and across these groupings, interventions were heterogeneous, impeding our ability to draw more specific conclusions per intervention type. However, we highlight overall implications for practice and research (Table 7).

**Table 7.** Implications for Practice, Policy, and Research.

Implications for Practice, Policy, and Research
• Culturally adapted psychological interventions can be effective for reducing a variety of symptoms of psychological distress in people with R/AS. • Cultural adaptations beyond increasing access to psychological interventions should be included in clinical practice, such as inclusion of cultural conceptualizations of distress, somatization of psychological symptoms, and incorporation of cultural practices, rituals, and beliefs through adaptation of goals, methods, and content. • Further testing of culturally adapted interventions is required to improve knowledge of effective and acceptable approaches across a variety of contexts and intervention intensities. • Investigation of patient acceptability of psychological interventions needs to be prioritized. • Investment is needed for increasing the availability of different intervention protocols, including harnessing the use of lay workers and brief interventions. • Funding should *not* be de-prioritized for CBT-based interventions despite the increase in resource needs, as these remain the most consistently effective.

CBT = cognitive behavioral therapy; R/AS = refugees and asylum seeker status.

First, intervention setting varied across groups, with half *transdiagnostic interventions* taking place in refugee camps, compared to all community-based interventions recorded in *CBT-based interventions*. Second, nearly all *transdiagnostic interventions* were provided by lay facilitators, compared to nearly all *CBT-based interventions* and *other psychotherapies* provided by trained professionals. Mixed results observed across transdiagnostic approaches may therefore reflect intervention timing (e.g., before established safety) and/or therapist competency effects. Third, majority of *CBT-based interventions* and *other psychotherapies* found intervention effects when compared to a non-active comparator (e.g., WLC), which may capture non-specific effects or reflect worsening symptoms resulting from being on a waitlist (Furukawa et al., 2014). From a pragmatic point, showing superior treatment effects over a WLC is still useful as in resource-constraint settings the default set-up is no treatment at all. If resources permit, future trials could also compare treatments with other active control conditions. Taken together, inclusion of setting, provider, and control type, as study moderators in future reviews could improve our understanding of which intervention works best in which context.

Despite methodological challenges of the literature, all reviewed *CBT-based interventions* and *other psychotherapies* studies provided evidence for the clinical effectiveness of culturally adapted interventions for a variety of psychological symptoms. Common cultural adaptations observed in interventions with moderate to large effect sizes included “language,” that is, use of cultural idioms of distress; “context,” modified timing, inclusion of family members and traditional foods; “content,” incorporation of religious or spiritual beliefs, emphasis on a somatic approach; “persons,” matched therapist to patient by sex, age, ethnicity; “metaphor,” use of culturally salient stories and images, “concept,” reframed to include familial/social relationships, and/or mind to body connection; “goals,” changed solution from individual to collective; and “method,” use of concrete strategies and reduced treatment components. An intervention approach recently gaining traction in clinical psychology is the focus on targeting distressing symptoms rather than complex diagnoses such as PTSD (Iyadurai et al., 2019), for example, targeting intrusive memory symptoms using a brief digitally supported (game-based) intervention distilled from experimental science (Lau-Zhu et al., 2019, 2021; Rackham & Lau-Zhu, 2021), including refugees (Kanstrup et al., 2021). Such approaches have potential for overcoming language barriers and stigma, as such, thoughtful consideration of cultural adaptations as discussed here will likely be crucial to optimize efficacy and acceptability for scalable implementation for digitally supported approaches, which were beyond the scope of this review.

Finally, assessment of acceptability was hindered by limited reporting in most studies. Acceptability refers to the emotional and cognitive experience of people receiving an intervention (Sekhon et al., 2017). People with R/AS often face external pressures which could impact treatment adherence (Semmlinger & Ehring, 2022). Nevertheless, very few studies explored acceptability via quantitative and qualitative methods, highlighting a need for more robust research in this area. Considerations to keep in mind when exploring acceptability research for people with R/AS include affective attitude, as well as perceived burden, usefulness, ethicality, and understanding of an intervention (Sekhon et al., 2017).

### Limitations

This review had several limitations. Inclusion of papers in English-limited access to peer-reviewed journals. Use of the EVM framework provided a systematic basis for identifying adaptations, but the framework has been criticized for an inherit assumption that all approaches require the same level of cultural adaptation (Heim & Kohrt, 2019). Mechanisms of action behind types of intervention (i.e., IPT, EMDR) vary greatly, and so may require different levels of adaptation. The “list like” approach to cultural adaptation used in the EVM framework, has led to past reviews regarding studies as “culturally adapted” based on minimal adaptation (Wright et al., 2020). To address this, we applied a stricter criterion to what constitutes a cultural adaptation and searched for evidence of best practice. Thus, unlike previous studies, we did not consider a study to be culturally adapted just simply by virtue of including R/AS populations (Wright et al., 2020).

We also excluded several studies using NET due to a lack of cultural adaptations beyond just translating. While NET is arguably developed for use with people with R/AS (Neuner et al., 2008; Neuner et al., 2004), there is scope for reporting of consideration and incorporation of cultural practices and traditions, including specific visuals and illustrations, as well as acknowledging historic trauma relevant to specific communities (Bedard-Gilligan et al., 2022)—features which were not specified in most of the excluded NET studies.

### Conclusion

Considering the number of ongoing armed conflicts around the world and the resulting increases in refugees and asylum seekers (UNCHR, 2022), the current review provides a timely synthesis of culturally adapted interventions for adults with R/AS to inform clinical practice. We considered interventions aimed at reducing a range of psychological symptoms more appropriate to people with R/AS, given the high rates of mental health burden and comorbidities in this population. Our focus was on “truly” cultural adaptations which extended beyond translations and access to consider fluid contexts that shape beliefs, values, and patterns of shared experience (Bernal et al., 2009; Heim et al., 2021). More high-quality cultural adaptations and robust trials of these are now needed to meet the pressing needs of people with R/AS around the globe.

## Supplemental Material

sj-docx-1-tva-10.1177_15248380241262262 – Supplemental material for Cultural Adaptations, Efficacy, and Acceptability of Psychological Interventions for Mental Health in Adults? with Refugees and Asylum-Seeker Status: A Systematic Review Supplemental material, sj-docx-1-tva-10.1177_15248380241262262 for Cultural Adaptations, Efficacy, and Acceptability of Psychological Interventions for Mental Health in Adults? with Refugees and Asylum-Seeker Status: A Systematic Review by Lianne McDermott, Ikra Hameed and Alex Lau-Zhu in Trauma, Violence, & Abuse
