# Supplementary material for: Cultural Adaptations, Efficacy, and Acceptability of Psychological Interventions for Mental Health in Adults with Refugees and Asylum-Seeker Status: A Systematic Review
Source: Trauma Violence Abuse. 2024 Aug 3;25(5):3758–76. doi: 10.1177/15248380241262262 (PMC11545127; doi:10.1177/15248380241262262)
Supplement: sj-docx-1-tva-10.1177_15248380241262262 – Supplemental material for Cultural Adaptations, Efficacy, and Acceptability of Psychological Interventions for Mental Health in Adults? with Refugees and Asylum-Seeker Status: A Systematic Review [file sj-docx-1-tva-10.1177_15248380241262262.docx]

**Appendix A: Search terms**

| *(refugee)) OR (asylum seeker)) OR (forcibly displaced))* |
| --- |
| *AND* |
| **"mental health" or "psychology" or "distress" or "depression" or "anxiety" or "emotional" or "trauma" OR "PTSD" OR "post-traumatic stress" OR "or "psychosis" OR "somatic' OR "transdiagnostic" or "psychiatric"** |
| *AND* |
| **"intervention" or "therapy" or "treatment" or "NET" or "CBT" or "EMDR" or "cognitive behavioural" or "counselling"** |
| *AND* |
| *(Randomized Controlled Trial)) OR (RCT))* |
| *NOT* |
| *(Children)) NOT (Adolescents)* |

**Appendix B:** *Intervention Groups*

| **Intervention Group** | **Description** | **Intervention’s Included** |
| --- | --- | --- |
| **Transdiagnostic Interventions** | Transdiagnostic approaches involves treatment of a range of symptoms and comorbid mental health symptoms. Approaches  vary with regards to key mechanisms and may borrow from and combine different treatment approaches (Uphoff et al., 2020). Interventions may include components such as psychoeducation, problem solving, cognitive restructuring, relaxation, behavioural activation, behavioural techniques. | Problem Management+  Common Elements Treatment Approach  Integrative Adaptive Therapy  Teaching Recovery Techniques |
| **CBT** | CBT can be defined as any intervention that conceptualises cognitions, particularly negative thoughts, and beliefs, and aims to reframe thinking styles as well as adapting behaviours that influence negative cognitions (Beck, 1979).  Trauma Focused CBT addresses cognitive, behavioural, and somatic symptoms relating to specific traumas.  NET is a type of CBT and focuses on processing a series of traumatic experiences via exposure and habituation. It involves a patient constructing of an autobiographical narrative (Schauer, 2011). | Culturally Adapted CBT  Cognitive Processing Therapy  Narrative Exposure therapy  Trauma Focused CBT  Transdiagnostic CBT |
| **Other Psychotherapies,** including EMDR, Third wave CBT approaches, interpersonal, cognitive analytic, humanistic, and other integrative therapies | Third Wave CBT involves conceptualisation of cognitions and emotions. Attention Is paid to the function of cognition (e.g. avoidance, suppression). Strategies Focus on changing thinking processes.  EMDR: an evidenced based trauma-focused treatment using bilateral simulations, initially theorised based on adaptive information processing, but developments suggest a working memory explanation. | EMDR:  Third Wave Approaches:  Acceptance and commitment therapy  Compassionate mind training  Mindfulness‐based therapy  Dialectic behaviour therapy  Interpersonal Psychotherapy |

**Appendix C: Further Information regarding risk of bias**

Regarding randomization bias, all but one study was categorised as “low risk”, with the remaining classified as “some concern” for inclusion of non-randomised participants in the intention-to-treat analysis (Shaw et al., 2019). Regarding recruitment bias specific to Cluster-RCTs, one study was categorised as “high risk” due baseline imbalances between clusters, and the recruitment process based on location (Green et al., 2021). For the domain of intended interventions, 94.4% of studies were assessed as “low risk”, due to the impossibility of blinding participants to intervention, combined with no serious faults reported. The cluster-RCT was categorised as “some concerns” due to reported differential intervention conditions and completion between groups. “Low risk” was described for the ‘missing data’ domain in 94.4% of studies, although significant missing data was reported in 50%, comparison between groups did not show significant differences between these data, and appropriate intent to treat analysis was utilised. The cluster-RCT was assessed as “some concerns”, for reporting baseline between-group differences that may have accounted for attrition and completion. For outcome measurement bias, all trials used self-report scales as outcome measures,

common in psychological interventions. Although 50% of studies reported independent outcome assessors blinded to participant intervention, the desirability bias from subjective self-report measures was unavoidable. Due to this inevitability, all studies were classified as “some concerns”. For the domain referring to result selection, the possibility of comparing results and methods of statistical analysis across studies with published protocols was possible in 50% of studies. A classification of “some concerns” was used to describe the remaining 50% of studies where protocols were not available or intended analysis was not reported prior to trial.

**Appendix D: Changes from protocol**

| **Protocol** | **Final Version** |
| --- | --- |
| Data Extraction: Cultural Adaptation- Initial plan to rate as culturally adapted versus not adapted in each domain as classified by EVM framework (e.g. one versus zero point). | Data Extraction: Cultural Adaptation- no longer quantifying level of cultural adaptation, instead describing nature and extent of adaptation, due to difficulties distinguishing between domains and comparing heterogenous interventions. |
| Data Selection: Any intervention with evidence of cultural adaptation | Data selection: Exclusion of interventions if only adaptation was to facilitate access without reference to culture |
| Data Selection: Exclusion criteria to include any RCT testing effects of intervention with R/AS | Data Selection: Exclude any study with less than 12 participants in treatment arm. |
| Supervisor listed as Dr Matthew Hotton | Supervisor listed as Dr Alex Lau Zhu |

**Appendix E:** *Cultural Adaptation according to the EVM (Bernal & Sáez‐Santiago, 2006)*

| **Intervention Group** | **Study ID*** | **Theoretical Underpinning** | **Formative Research** | ***Language*** | ***Persons*** | ***Metaphors*** | ***Concepts*** | ***Goals*** | ***Methods*** | ***Context*** | ***Content*** |
| --- | --- | --- | --- | --- | --- | --- | --- | --- | --- | --- | --- |
| Group 1: Transdiagnostic Approaches | Acarturk 2022 | Absent | Pilot trial with Arabic speaking Syrians, Free list interviews, key informant interviews, focus groups, and adaptation workshops. | Use of cultural idioms of distress, intervention delivered in native language by facilitator | Peer facilitators used, sex matched; Recommendations to facilitators to add suggestions of appropriate verbal and physical reinforcement in sessions. | Inclusion of metaphors, stories, and illustrations | Expand on examples of adversity to include familial relationships | Change solutions of goals from individual to others | Use of worksheets to simplify methods | Inclusion of a male case study; country specific adaptations | Addition of a session designed to enhance family engagement.  Remove yoga as a relaxing technique and replace with culturally appropriate suggestion |
|  | Bolton 2014 | Past study identified fear of attending public places due to fear of deportation (Abshir et al., 2010); Previous research indicated high prevalence of alcohol abuse in this population. | Literature review,  Qualitative studies with Burmese individuals. | Use of colloquial language; cultural idioms of distress;  Assessment tools translated and adapted | Peer facilitators trained for purpose of study. Matched therapist to patient -shared cultural backgrounds | Utilisation of Burmese folktales, personal anecdotes, and local expressions or adages to convey key principles. | NR | Building on existing strengths e.g. support and family and community, meditation; Encouragement of skills in line with Burmese traditions | NR | Family members invited to sessions; setting adapted | Inclusion of alcohol use and trauma components |
|  | Bryant 2022 | Absent | Pilot trial with Arabic speaking Syrians, Free list interviews, key informant interviews, focus groups, and adaptation workshops. | Not all outcome measures adapted, but all translated.  Use of culturally equivalent idioms of distress | Peer facilitators used. Recommendations to facilitators to utilise appropriate reinforcement in sessions. | Inclusion of metaphors, stories, and illustrations | Expand on examples of adversity to include familial relationships | Change solutions of goals from individual to others | Use of worksheets to simplify methods | Inclusion of a male case study; country specific adaptations | Addition of a session designed to enhance family engagement.  Remove yoga as a relaxing technique and replace with culturally appropriate suggestion |
|  | De Graff 2020 | Absent | Qualitative interviews with Syrian refugees and professionals, and cognitive testing of the literally translated manual.  Measures pilot tested. | Adapted and translated measures administered as interviews; Use of culturally equivalent idioms of distress | Peer facilitators used  Recommendations to facilitators to utilise appropriate reinforcement in sessions. | Inclusion of metaphors, stories, and illustrations | Expand on examples of adversity to include familial relationships | Change solutions of goals from individual to others | Use of worksheets to simplify methods | Case examples rephrased to fit Syrian context | Core components of the PM+ intervention considered and retained |
|  | De Graff 2023 | Absent | Literature review, stakeholder engagement, rapid qualitative assessments, literal translation, cognitive interviews, adaptation workshops and finalisation of the manuals. | Adapted and translated measures administered as interviews; Use of culturally equivalent idioms of distress | Peer facilitators used  Recommendations to facilitators to utilise appropriate reinforcement in sessions. | Inclusion of metaphors, stories, and illustrations | Expand on examples of adversity to include familial relationships | Change solutions of goals from individual to others | Use of worksheets to simplify methods | Case examples rephrased to fit Syrian context | Core components of the PM+ intervention considered and retained |
|  | Hasha 2022 | Absent | A consulting group of male and female Syrians. Arabic background of resource person. | Interpreters used; vocabulary discussed prior, outcome measures translated and adapted | NR | Relevant examples adapted to suit adult Syrians. | NR | Priority for importance of socializing | Homework tasks adapted to suit adult Syrians | Gender-segregated or age-segregated groups; the frequency and timing of intervention; coordinated childcare; text reminders; dress code | NR |
|  | Greene 2021 | CCD informed adaptation: msongo wa mawazo (stress, too many thoughts), huzuni (deep sadness), and hofu (fear) | A desk review; study site visit; formative qualitative research; and expert consultation. | Intervention in native language’ outcome measures translated and adapted; culturally salient examples; strength langauge used to describe mental health | Lay providers trained for purpose of study | NR | NR | NR | Emphasis on empowerment; emphasis on cognitive processing | Sessions condensed; age composition matched and homogenised | Added an intimate partner violence intervention component |
|  | Tay 2020 | Review of cultural idioms of distress and mental health symptoms informed adaptation | Focus groups,  Epidemiological study, desk review of local terminologies,  qualitative assessments. Reports by key informant interviews with community members/clinicians. | Translated material (with dialects) . Translated and adapted outcome measures; consideration of cultural idioms of distress | Lay Counsellors trained for purpose of study | Inclusion of metaphors including materials of specific cultural relevance  Culturally relevant images | Use of holistic insights into maladaptive emotions and behaviours that have caused psychosocial disruptions | Goals established around the impact of displacement on individual and the collective level | Materials using cultural expressions based on the five ADAPT pillars incorporated to increase comprehensibility of the therapy content | NR | NR |
| Group 2: CBT | Eskici 2023 | CDD informed adaptation e.g. Cultural syndrome that is called ***Noba,*** which indicates an episode of panic and loss of consciousness after being in severe distress. | Literature review, interviews, focus groups, consultation of Syrian/Turkish psychiatrists to identify culturally informed language and intervention. | Bilingual therapists;  Outcome measures translated.  Cultural idioms of distress utilised. | NR | Practices, idioms, proverbs, analogies, metaphors into account | Explanatory model bridging shaped treatment in line with present culture’s approach toward mind and body. | NR | NR | Sessions condensed | A somatic and sensorial approach was emphasized, as well as emotion regulation, mindfulness, stretching, and adaptive imagery |
|  | Hijazi 2014 | Absent | No information | Bilingual therapist,  Narrative provided in Arabic; measures adapted and translated | NR | NR | Constructs of theoretical model used in treatment were culturally relevant | NR | NR | Condensed therapy. Setting adapted. Option of religious setting. | NR |
|  | Hinton 2005 | CCD informed adaptation: neck and orthotactically focused panic attacks specific to this population | Preliminary studies of trauma-related disorder among Cambodian and Vietnamese refugees. | Therapy in native language;  Culturally specific measure of psychopathology; cultural idioms of distress | Multilingual, multicultural psychiatrist | Culturally appropriate visualisation incorporated into intervention | TCMIE model utilised, emphasising somatic approach.  Psychoeducation included the difference between PTSD symptoms and culturally distinct symptoms. | Focus on reduced symptoms of culturally salient symptoms of distress | Adapted procedures for achieving the treatment goals e.g., teaching cognitive flexibility through visualisation | NR | Emphasis on Buddhist principles e.g. mindfulness,  Visualisation included enacting analogous rotational movements,  Psychoeducation around culturally relevant somatic symptoms |
|  | Hinton 2005 | CCD informed adaptation; A theoretical model of panic and | Previous pilot studies. | Therapy in native language, culturally sensitive measures and idioms of distress | Multilingual, multicultural psychiatrist | Culturally relevant idioms, symbols, and metaphors | A theoretical model of the generation of PTSD among Cambodian refugees | Focus on increasing emotional regulation capacity and decreasing PAs, including orthostatic panic | Adapting procedures for achieving the treatment goals | NR | Emotion regulation  Psychoeducation on culturally specific somatic symptoms and cognitions.  Yoga‐like stretching linked to self‐images of flexibility |
|  | Kananian 2020 | CCD informed adaptation; Promotion of health behaviours due to stigma theory | Pilot study, focus groups with male and female Afghan refugees conducted | Bilingual therapist  Translated materials; cultural idioms of distress (syndromes and symptoms) | NR | Culturally appropriate guided imagery | explanatory models and symptom descriptions were adapted to the cultural context |  | The training was simplified. Relevant case examples and problem-solving exercises. | Gender homogenous groups, condensed sessions | Enhanced problem-solving element; each session included yoga and stretching exercises. |
|  | Shaw 2019 | CCD informed intervention | Qualitative study of mental health needs. Review of literature on mental health needs in this population. | Outcome measures adapted and culturally sensitive; therapy in native language; Cultural idioms of distress; Presented as mental health promotion and wellbeing due to stigma | Afghan therapist without formal training, trained for purpose of study | Culturally and religiously appropriate symbols and imagery | Focus on somatic symptoms and emotion regulation | NR | NR | Young children allowed to sessions;  snacks provided | Inclusion of visualisation exercise  Components focused on dysphoric worry; Inclusion of religious and spiritual practice |
| Group 3: Other Psychotherapies | Acarturk 2015 | Stigma theory informed adaptations | No information | Interpreters,  Outcome measures translated and adapted | Therapist and/or interpreters were matched to patient via sex | Religious imagery used | NR | NA to for all empty (then add note | NR | Introductory sessions to key community members; Setting and timing of therapy adapted; childcare provided | Additional psychoeducation |
|  | Acarturk 2016 | Stigma theory and MHL informed adaptations e.g. past study identified 0.5 psychiatrists and no psychologist per 100,000 population in Syria (IASC, 2007). | Literature review,  acceptability and efficacy pilot RCT. | Interpreters,  Outcome measures translated and adapted | Therapist and/or interpreters were matched to patient via sex | Religious imagery used | NR | NR | NR | Introductory sessions to key community members; Setting and timing of therapy adapted; childcare provided | Additional psychoeducation |
|  | Aizik-Reebs 2021 | Absent | Modified based on feedback from researchers, clinicians, asylum seekers, and via cognitive interviews with asylum seekers. | Linguistic translation (Tigrinya), Outcome measures translated and adapted. Use of culturally appropriate idioms of distress | Cultural mediators included, therapists were sex matched | NR | NR | NR | Fewer elements, simplified language, concrete strategies | Setting adapted.  Participants offered free childcare | Eritrean food brought into sessions;  Cognitive elements of MBCT (e.g., reducing believability in negative thoughts) not included |
|  | Meffert 2014 | Absent | Review of data from needs assessment and adaptation study, literature review, and discussion with community and local partners | Outcome measures translated and adapted; therapy in native language | Sudanese community therapists trained for purpose of study (ethnicity matched) | NR | NR | NR | NR | Condensing the intervention; setting adapted | The IPT foci were limited to interpersonal disputes, role transitions, or grief. |
